# Supplementary figures and images for: Virulence profiles of some Pseudomonas aeruginosa clinical isolates and their association with the suppression of Candida growth in polymicrobial infections
Source: PLoS One. 2020 Dec 8;15(12):e0243418. doi: 10.1371/journal.pone.0243418 (PMC7723275; doi:10.1371/journal.pone.0243418)

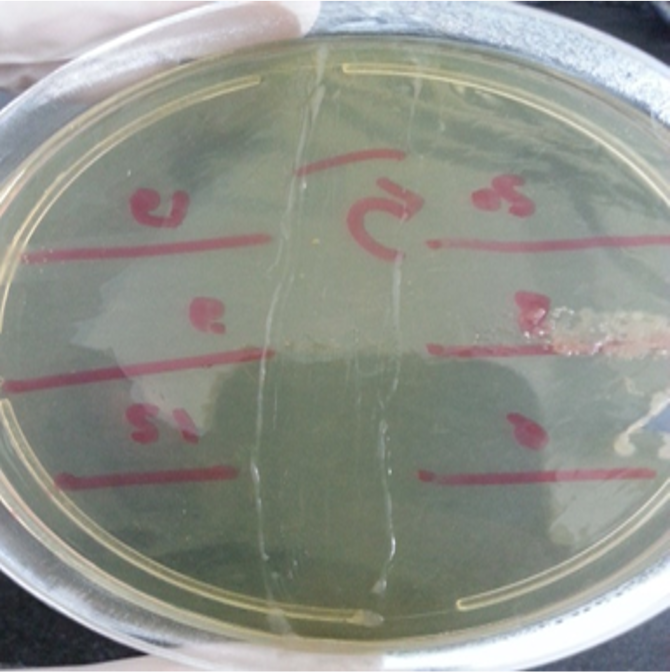

Supplement: S1 Raw images — (ZIP) [file pone.0243418.s001.zip › Figure 1A.tif]

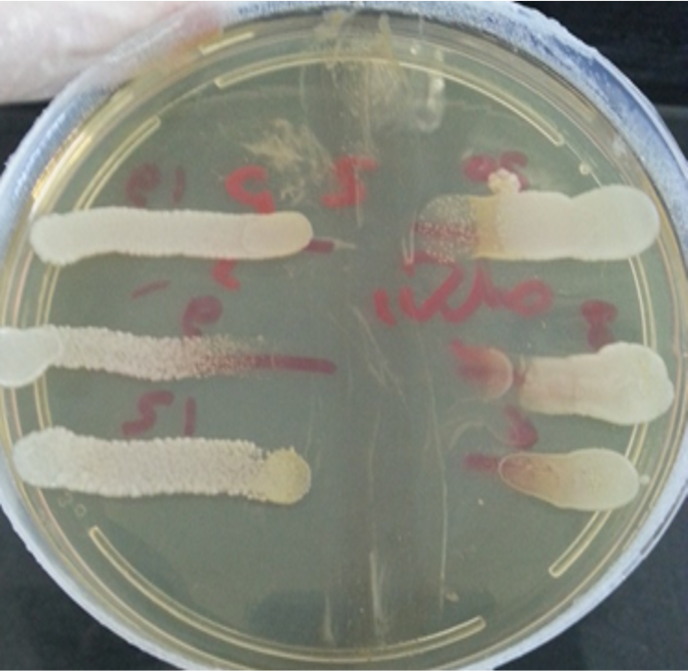

Supplement: S1 Raw images — (ZIP) [file pone.0243418.s001.zip › Figure 1B.tif]

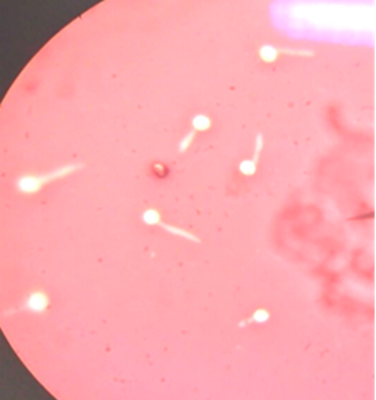

Supplement: S1 Raw images — (ZIP) [file pone.0243418.s001.zip › Figure 2A.tif]

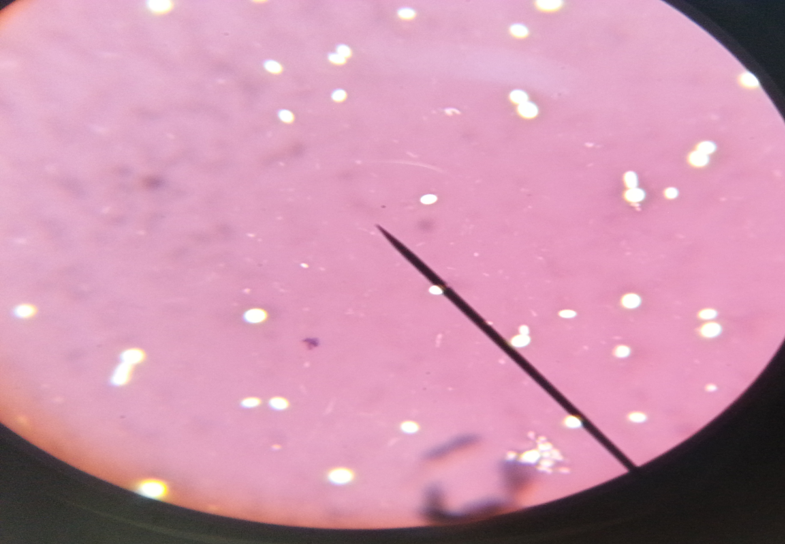

Supplement: S1 Raw images — (ZIP) [file pone.0243418.s001.zip › Figure 2B.tif]

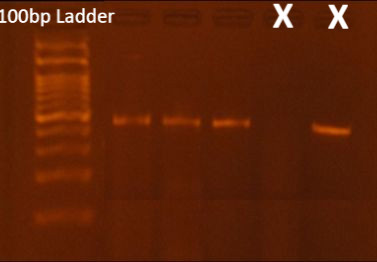

Supplement: S1 Raw images — (ZIP) [file pone.0243418.s001.zip › Figure 3A phzP.jpg]

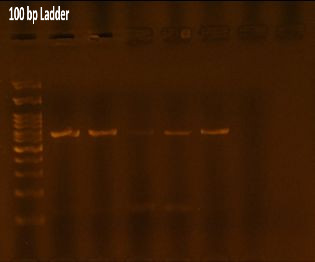

Supplement: S1 Raw images — (ZIP) [file pone.0243418.s001.zip › Figure 3A phzS.jpg]

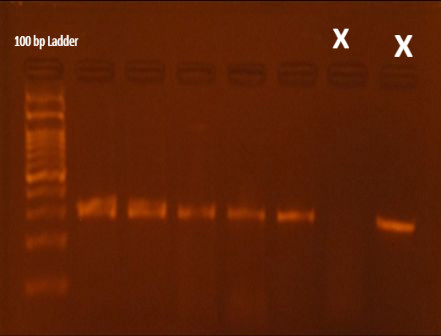

Supplement: S1 Raw images — (ZIP) [file pone.0243418.s001.zip › Figure 3A. phzM.tif.jpg]

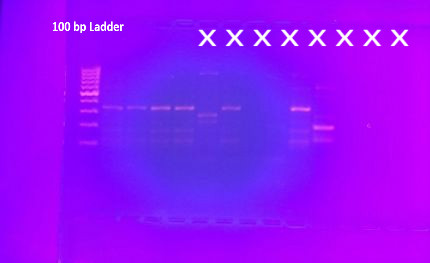

Supplement: S1 Raw images — (ZIP) [file pone.0243418.s001.zip › Figure 3B Alg D.jpg]

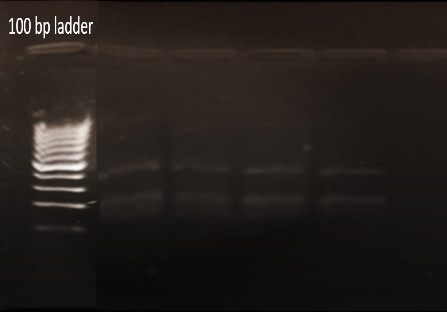

Supplement: S1 Raw images — (ZIP) [file pone.0243418.s001.zip › Figure 3B exoS.jpg]

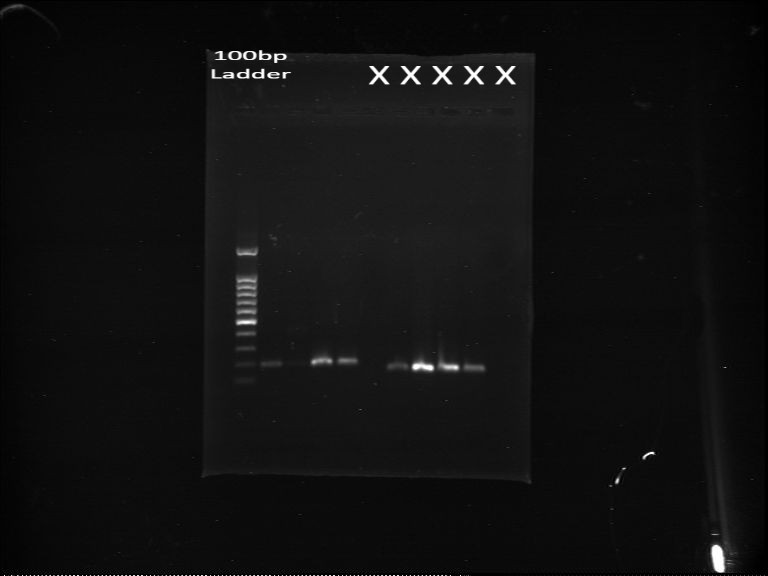

Supplement: S1 Raw images — (ZIP) [file pone.0243418.s001.zip › Figure 3B LasB.jpg]

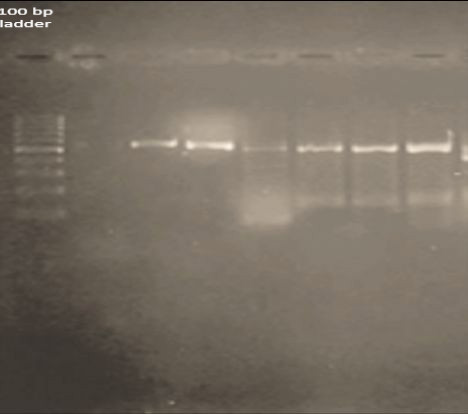

Supplement: S1 Raw images — (ZIP) [file pone.0243418.s001.zip › Figure 3B plcH.jpg]

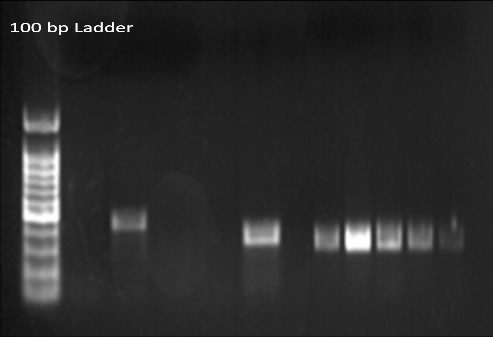

Supplement: S1 Raw images — (ZIP) [file pone.0243418.s001.zip › Figure 3B plcN.jpg]

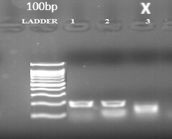

Supplement: S1 Raw images — (ZIP) [file pone.0243418.s001.zip › Figure 3B ToxA.jpg]

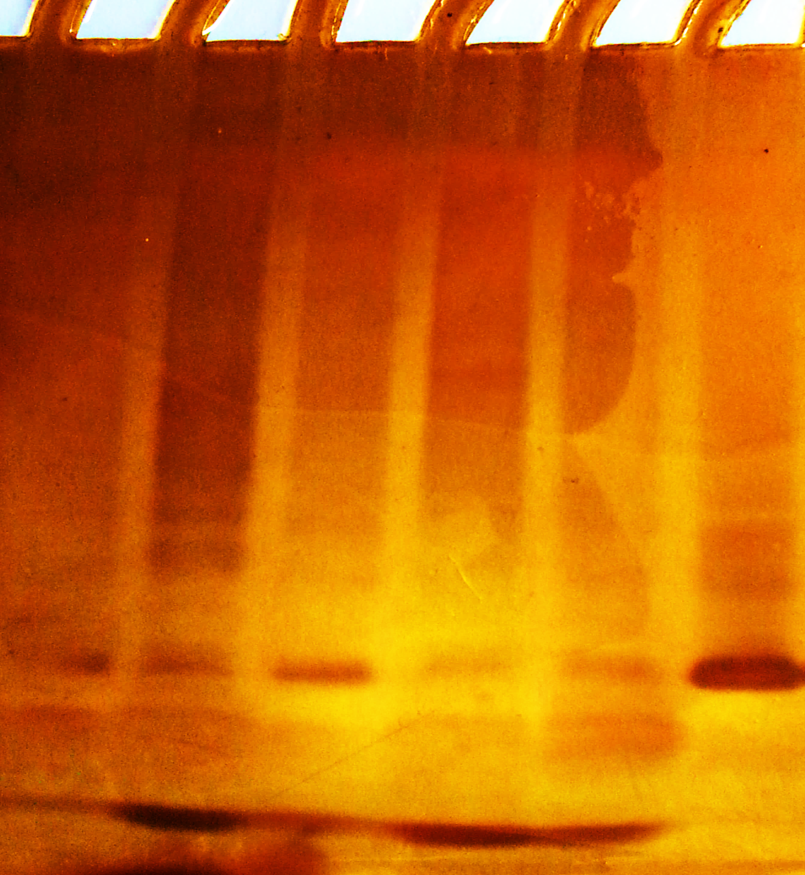

Supplement: S1 Raw images — (ZIP) [file pone.0243418.s001.zip › Figure 4..tif]

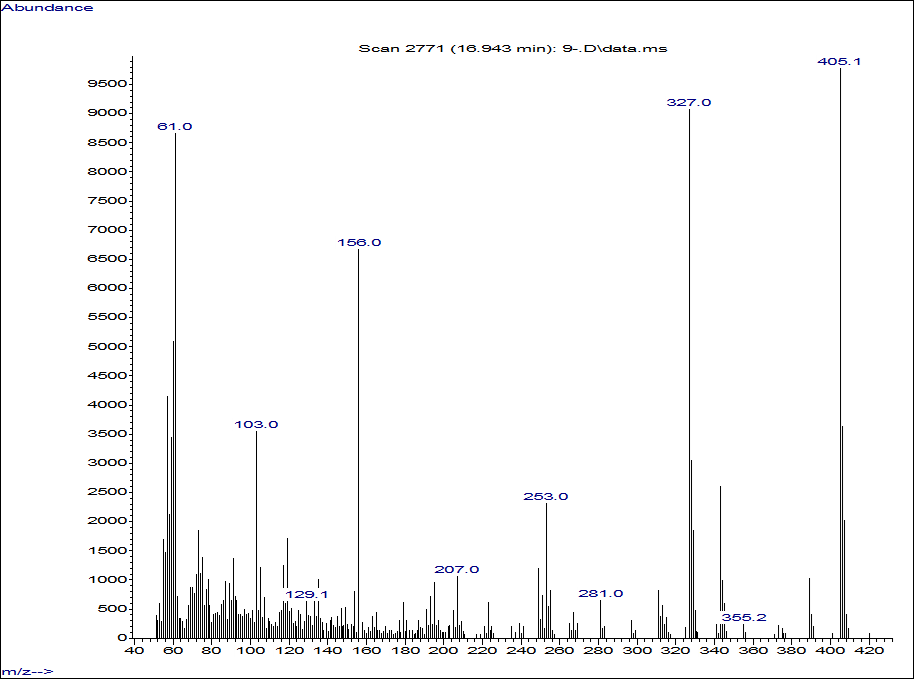

Supplement: S1 Raw images — (ZIP) [file pone.0243418.s001.zip › Figure 5.tif]

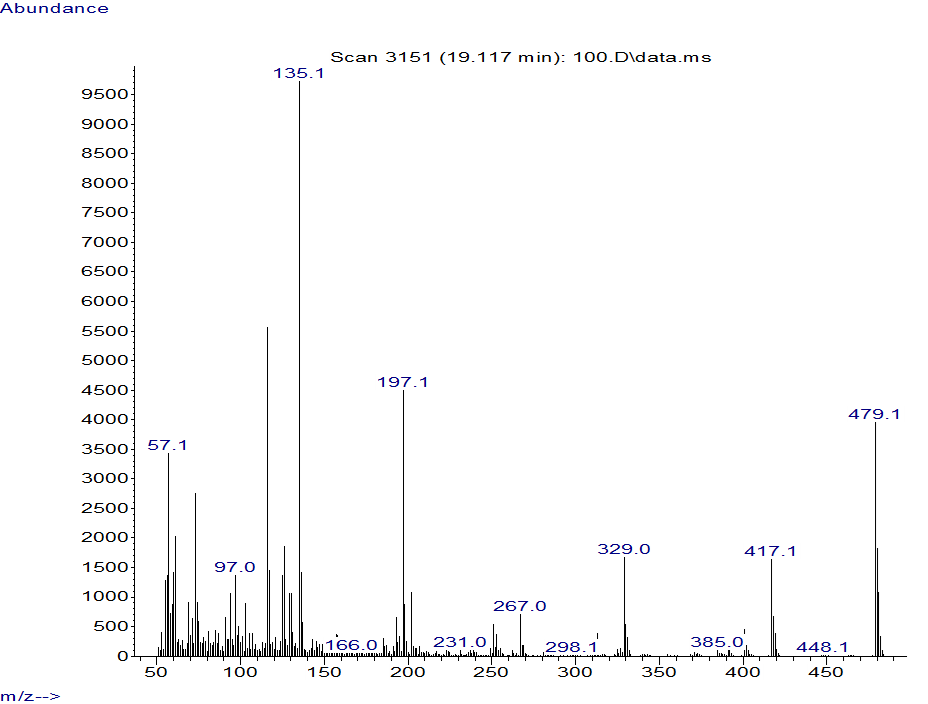

Supplement: S1 Raw images — (ZIP) [file pone.0243418.s001.zip › Figure 6.tif]

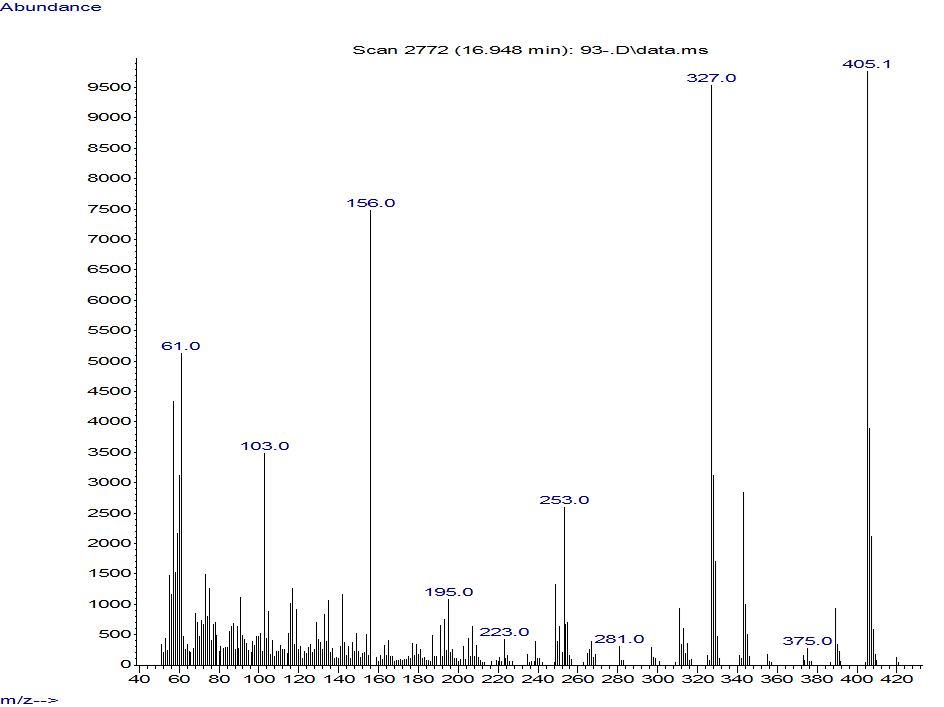

Supplement: S1 Raw images — (ZIP) [file pone.0243418.s001.zip › Figure 7.tif]

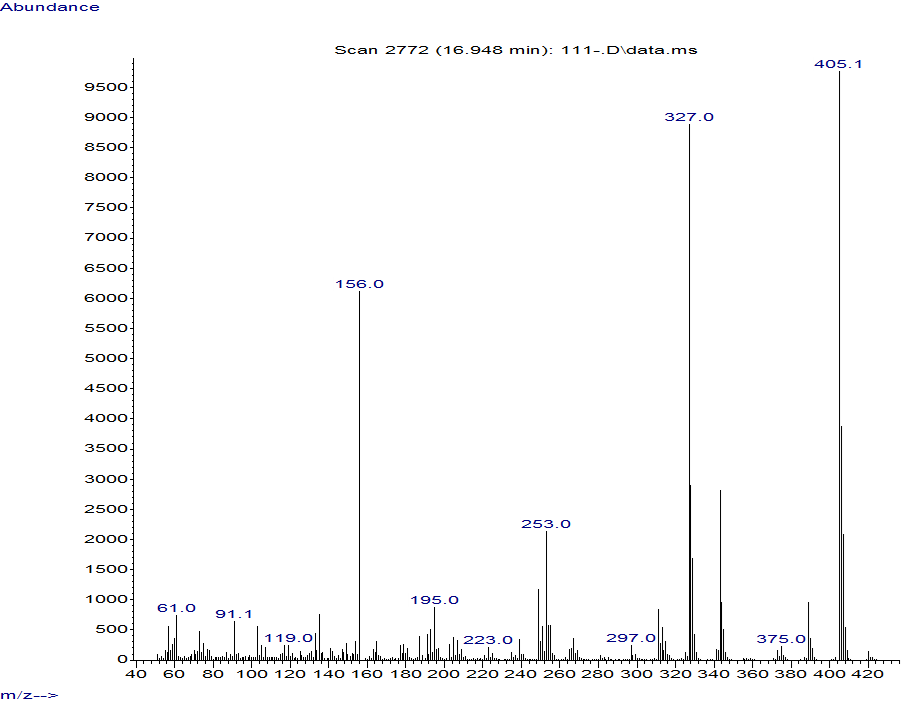

Supplement: S1 Raw images — (ZIP) [file pone.0243418.s001.zip › Figure 8.tif]

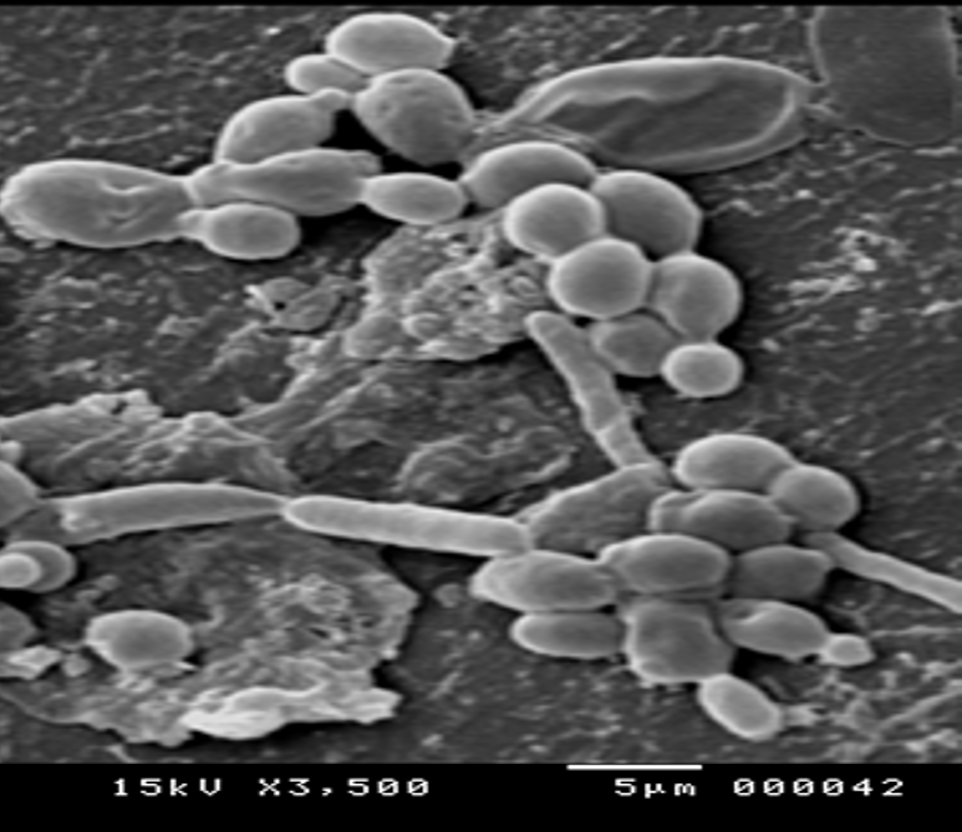

Supplement: S1 Raw images — (ZIP) [file pone.0243418.s001.zip › Figure 9 A.tif]

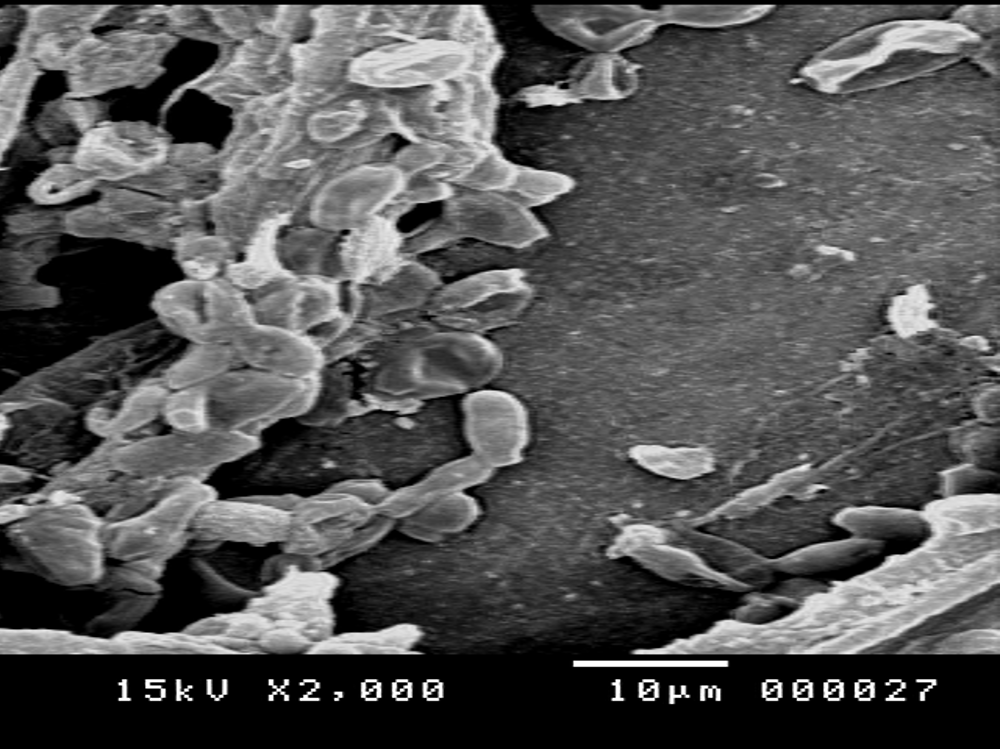

Supplement: S1 Raw images — (ZIP) [file pone.0243418.s001.zip › Figure 9 B.tif]
