# Supplementary material for: Virulence profiles of some Pseudomonas aeruginosa clinical isolates and their association with the suppression of Candida growth in polymicrobial infections
Source: PLoS One. 2020 Dec 8;15(12):e0243418. doi: 10.1371/journal.pone.0243418 (PMC7723275; doi:10.1371/journal.pone.0243418)
Supplement: Attachment — Submitted filename: Response to Reviewers.docx [file pone.0243418.s002.docx]

Reviewer #1:

This manuscript demonstrates natural phenazine compounds produced by Pseudomonas, and describe anticandidal activity against 3 different Candida spp. Then they also checked the effect on hyphae production and some

virulence gene. Finally they analyzed the fatty acids by GC-MS as methyl ester derivatives. Manuscript needs more refinements in addition to several issues which need to be addressed.

|  | Comments |
| --- | --- |
| 1) Candida should be mentioned in ilatics in line no. 2. 2) The spelling of C. krusi is wrong, it should be C. krusei. 3) Please be accurate in writing the degree centigrade unit. 4) Candidal can be replaced by Candida in line no. 99. 5) "and" before 42°C can be removed in line no. 113. 6) It will be more appropriate to write Pseudomonas aeruginosa as P. aeruginosa instead of Ps. aeruginosa in methods section. 7) Authors can also use one more method for determining anticandidal activity. 8) Please convert rpm into g in line no. 145. 9) There is no uniformity of using units as somewhere written μl or μL. Please correct this throughout the manuscript. 10) Please mention the full name of light microscope company mentioned in line no. 152. Same should be written in line no.167. 11) Please ensure to write either hour or hours throughout the manuscript like in line no. 153, hours is written and in line no.156, it is written hrs. 12) figure 3B is written in line no. 228, authors have written as Figure throughout the manuscript, please be uniform. 13) Authors have used only single method for germ tube formation. There are many other hypha inducing media for studying the effects on germ tube formation. I would recommend the authors to use one more method to validate the observations. 14) Please mention the reference for method used for SEM, generally 4% glutaraldehyde is used for fixing the cell. After drying with ethanol, TMS/HMDS is used after drying and before sample mounting. 15) In section, 2.4, the method used for DNA isolation is unclear. Please mention the protocol used briefly.   16) Phospholipases enzyme are also secreted by C. albicans to degrade the host tissues (https://pubmed.ncbi.nlm.nih.gov/9467900/). So, it is unclear that how the phospholipases of one species affect the  phospholipases of another species belonging to same class. Please put some more evidences to support you findings.   16) Please recheck the references no. 1 and 44. 17) Figures resolution and clarity should be ensured. Some words are blurred due to over zooming.   19) Authors have mentioned only about the antagonistic relation of P.aeruginosa and C. albicans where as this is not always the case. Please refer to this article Dhamgaye, S., Qu, Y., & Peleg, A. Y. (2016). Polymicrobial infections involving clinically relevant Gram-negative bacteria and fungi. Cellular microbiology, 18(12), 1716–1722. https://doi.org/10.1111/cmi.12674  (Studies have showed both antagonistic and synergistic interactions, depending on diverse environmental factors, timing of interaction, and growth state at the time of interaction. A lower concentration of phenazines impaired hyphal growth of C. albicans and more importantly switched fungal respiration to fermentation leading to the production of ethanol, glycerol, and acetate by C. albicans in glucose containing media (Morales et al., 2013). It was shown that C. albicans ethanol production not only influenced biofilm maturation but also promoted more phenazine production by P. aeruginosa through WspR‐dependent activation of Pel exopolysaccharide (Chen et al., 2014). The spectrum of P. aeruginosa phenazines produced was in favor of those most effective against fungal cells and led to greater production of ethanol by C. albicans , forming a feedback loop driving the polymicrobial interaction towards the protection of P. aeruginosa (Chen et al., 2014).It has now been shown that  Candida colonization of the respiratory tract may promote the development of pseudomonal VAP and has been associated with the presence of multidrug‐resistant bacteria (Azoulay et al., 2006; Hamet et al., 2012). The laboratory studies characterizing the diverse interactions between Candida and Pseudomonas highlight the real complexities of their interaction, and questions still remain as to how they apply during human infection.  20) The manuscript is not thoroughly written, it need lots of language and grammar refinements.  21) There is nothing written about the statistical significance (p value) of the work.  22) The conclusion is not represented very well. Authors can rewrite this section.  23) The work is good but lacks the novelty as many previous studies have reported the interaction of both species in  polymicrobial infections. | Corrected  Corrected  Corrected  Corrected  Corrected  Corrected  Line 140 page 5  It is modified  All units were revised  It was added  Corrected  Corrected  Line 149 page 5  Line 161 page 6  Reference is added  It was rewritten  Line 58 to 71 page 14 and 15  References checked and modified  The original figure for blurred are placed  It was added line 196 to 200 page 7 and 8.  It was revised  It was added  It was rewritten |
| Reviewer #2: Major comments:  The authors should clarify the link between P. aeruginosa virulence and anticandidal activity in relation to clinical severity in patients. Did the strains with potent anticandidal activity show clinical manifestations in patients specific to Pseudomonas such as (pneumonia, urinary infections or wound infections) or there was no link. Please clarify and include this in discussion section. This is important since both pseudomonas and candida are opportunistic pathogens and they are part of normal flora in healthy individuals.  Minor Comments:  1-The authors should list the site of lesions for all isolated strains and clinical diseases of affected patients if there is any.  2-Conclusion should be expanded to highlight the significance of the study. | It was clarified through the manuscript  They were listed in the table 2  Conclusion was rewritten |

All changes were highlighted in the revised manuscript.
